# Phylogeography of *Francisella tularensis* subsp. *holarctica*, Europe

**DOI:** 10.3201/eid1802.111305

**Published:** 2012-02

**Authors:** Miklós Gyuranecz, Dawn N. Birdsell, Wolf Splettstoesser, Erik Seibold, Stephen M. Beckstrom-Sternberg, László Makrai, László Fodor, Massimo Fabbi, Nadia Vicari, Anders Johansson, Joseph D. Busch, Amy J. Vogler, Paul Keim, David M. Wagner

**Affiliations:** Hungarian Academy of Sciences, Budapest, Hungary (M. Gyuranecz);; Northern Arizona University, Flagstaff, Arizona, USA (D.N. Birdsell, J.D. Busch, A.J. Vogler, P. Keim, D.M. Wagner);; Bundeswehr Institute of Microbiology, Munich, Germany (W. Splettstoesser, E. Seibold);; Translational Genomics Research Institute, Phoenix, Arizona, USA (S.M. Beckstrom-Sternberg, P. Keim);; Szent István University, Budapest (L. Makrai, L. Fodor);; Istituto Zooprofilattico Sperimentale della Lombradia e dell’Emilia Romagna, Pavia, Italy (M. Fabbi, N. Vicari);; Umeå University, Umeå, Sweden (A. Johansson)

**Keywords:** Francisella tularensis subsp. holarctica, bioterrorism, phylogeography, SNP, canSNP, zoonoses, Europe

## Abstract

*Francisella tularensis* subsp. *holarctica* isolates from Austria, Germany, Hungary, Italy, and Romania were placed into an existing phylogeographic framework. Isolates from Italy were assigned to phylogenetic group B.FTNF002–00; the other isolates, to group B.13. Most *F. tularensis* subsp. *holarctica* isolates from Europe belong to these 2 geographically segregated groups.

*Francisella tularensis* is the etiologic agent of tularemia and a highly virulent category A biothreat agent (*1**,**2*). The most widely distributed subspecies is *F. tularensis* subsp. *holarctica*, which is found throughout much of the Northern Hemisphere and is the only subspecies found in Europe (*3*). Despite its wide geographic distribution, *F. tularensis* subsp. *holarctica* contains low genetic diversity, which indicates recent emergence (*4*). A recent global phylogeographic analysis (*5*), and several subsequent analyses (*6**–**9*), assigned most isolates from Europe to 2 phylogenetic groups: B.FTNF002–00 and B.13 (includes multiple subclades descended from branch B.13 [*5**,**6**,**8*]; branch and subclade nomenclature from [*5*] has been shortened by removing Br and extra 0s from individual branch and subclade names). These groups appear to be geographically segregated: only isolates from B.FTNF002–00 have been reported from the western European countries of Spain, France, and Switzerland, whereas B.13 is the only or dominant type reported from the Czech Republic, Finland, Georgia, Russia, Slovakia, and Ukraine (*5**–**9*). We provide additional information about the geographic distribution of these 2 groups using existing phylogenetic signatures (*5**,**8*) to place 45 isolates from Austria, Germany, Hungary, Italy, and Romania (Table A1) into the existing global phylogeographic framework.

## The Study

**Figure 1:**
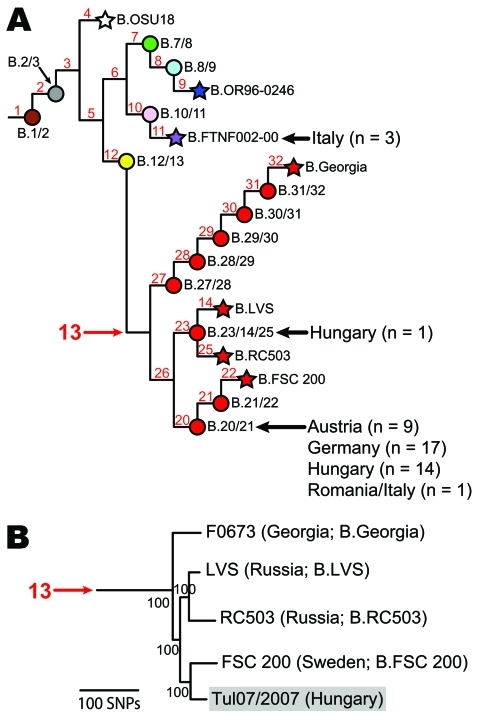
Existing phylogeny of *Francisella tularensis* subsp. *holarctica*. A) Single nucleotide polymorphism (SNP)–based phylogeny of *F. tularensis* subsp. *holarctica* derived from previous studies (*5**,**6**,**8*). Terminal subgroups representing sequenced strains are shown as stars, and intervening nodes representing collapsed branches are indicated by circles. Subclades within group B.13 are depicted in red. Isolates from Austria, Germany, Hungary, Italy, and Romania (n = 45) were assigned to existing subclades (black arrows) by using existing canonical SNP assays (*5**,**8*). B) Maximum parsimony phylogeny constructed by using SNPs discovered from 6 *F. tularensis* whole-genome sequences, including 5 strains from group B.13 and an outgroup strain, OSU18 (not shown). This phylogeny was rooted by using OSU18, and bootstrap values were based on 1,000 simulations by using a heuristic search. The newly sequenced Hungarian strain (Tul07/2007) is highlighted in gray.

**Figure 2:**
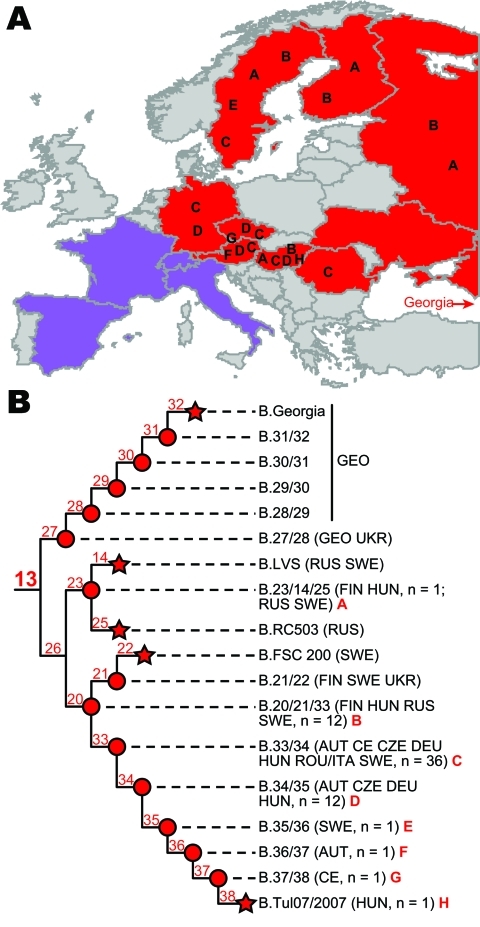
Detailed geographic distribution and phylogeny of *Francisella tularensis* subsp. *holarctica* subclades within group B.13. A) Countries from which groups B.13 and B.FTNF002–00 have been reported. Countries of origin for isolates assigned to select subclades within group B.13 are indicated by the letters A–H. Red and purple shading indicates the known geographic distributions of groups B.13 and B.FTNF002–00, respectively, in this and previous studies (*5**–**9*). The country of Georgia, which also contains isolates from group B.13 but is not depicted in the map, is indicated by red text and a red arrow pointing toward its location. Isolates assigned to other phylogenetic groups within *F. tularensis* subsp. *holarctica* have been reported from some of these countries (*5**,**8*), but most isolates from these countries are from groups B.13 and B.FTNF002–00. B) Single nucleotide polymorphism–based phylogeny of previously (*5**,**6**,**8*) and newly identified subclades within the B.13 group of *F. tularensis* subsp. *holarctica*. Terminal subgroups representing sequenced strains are shown as stars, and intervening nodes representing collapsed branches are indicated by circles. The countries of origin for isolates assigned to each subclade are indicated: AUT, Austria; CE, central Europe, unknown country; CZE, Czech Republic; DEU, Germany; FIN, Finland; GEO, Georgia; HUN, Hungary; ITA, Italy; ROU, Romania; RUS, Russia; SWE, Sweden; UKR, Ukraine). For mapping purposes, letters are assigned to a previously identified subclade that contains a new isolate from Hungary now assigned to that subclade (A) and newly identified subclades (B–H). The number of isolates listed for each subclade refers only to isolates examined directly in this study (Table A1).

All of the isolates were assigned to group B.FTNF002–00 or to group B.13. All 3 isolates from Italy were assigned to group B.FTNF002–00 (Figure 1, panel A). Although the sample size was small, these isolates were obtained in 3 different years (Table A1), which suggests that this group is ecologically established in Italy. These results increase the known geographic distribution of this group, which appears to be the dominant clone in western Europe (Figure 2, panel A, purple shading). All 42 isolates from Austria, Germany, Hungary, and Romania were assigned to group B.13 (Figure 1, panel A), further demonstrating that B.13 is the most prevalent group of *F. tularensis* subsp. *holarctica* in central and eastern Europe (Figure 2, panel A, red shading). Within group B.13, one isolate from Hungary was assigned to subclade B.23/14/25 (Figure 1, panel A); isolates from Finland, Russia, and Sweden were previously assigned to this subclade (*6**,**8*) (Figure 2, panel B). However, the other 41 isolates were assigned to subclade B.20/21 (Figure 1, panel A).

We identified new genomic signatures to provide increased genetic resolution within subclade B.20/21. Next-generation sequencing technology (Illumina Inc., San Diego, CA, USA) was used to sequence the genome of an isolate from Hungary (Tul07/2007, GenBank accession no. SRX025133) assigned to subclade B.20/21. Putative single nucleotide polymorphisms (SNPs) were identified in the resulting sequence and the genomes of 4 other strains previously assigned to group B.13 (LVS, AM233362.1; FSC 200, AASP00000000; RC503, SRX000104; Georgia F0673, SRX025885) by using an existing bioinformatics pipeline (*5*). The more distantly related strain OSU18 (CP000437.1) genome was also included as an outgroup. A maximum-parsimony tree was constructed by using the resulting ≈700 putative SNPs and PAUP 4.0b10 software (Sinauer Associates, Inc., Sunderland, MA, USA) (Figure 1, panel B). Most of the putative SNPs separated OSU18 from the B.13 strains (data not shown), but the remaining putative SNPs provided resolution among the B.13 strains, including 20 putative SNPs specific to the branch leading to the strain from Hungary (Figure 1, panel B). Consistent with previous analyses (Figure 1, panel A), the strain from Hungary clustered as a sister taxon to strain FSC 200 (Figure 1, panel B).

To show additional phylogenetic structure within subclade B.20/21, we designed genotyping assays targeting the 20 putative SNPs along the branch leading to the strain from Hungary (Figure 1, panel B) and screened them across 64 isolates assigned to subclade B.20/21. This analysis included the 41 isolates from Austria, Germany, Hungary, and Romania, as well as 23 additional isolates from central Europe, the Czech Republic, Finland, Russia, and Sweden that were previously assigned to this subclade (*6**,**8*) (Table A1). The assays were constructed and performed as described (*5*) by using an annealing temperature of 60°C. All 20 SNPs were laboratory confirmed, and 52 of the isolates were assigned to 6 new subclades (B.33/34, B.34/35, B.35/36, B.36/37, B.37/38, and B.Tul07/2007); the 12 other isolates remained in the basal subclade, now identified as B.20/21/33 (Figure 2, panel B; Table A1). Information about assays targeting canonical SNPs for the branches leading to the 6 new subclades are presented in the Table.

## Conclusions

Our results are consistent with complex dispersal patterns within the B.13 group of *F. tularensis* subsp. *holarctica*. Several of the B.13 subclades identified in this study are broadly distributed throughout central and eastern Europe (Figure 2, panel A), including subclades B.20/21/33, B.33/34, and B.34/35. All of the new subclades containing >1 isolate have representatives from multiple countries (Figure 2, panel B). Other previously identified B.13 subclades, including B.27/28, B.LVS, B.23/14/25, and B.21/22 are also broadly distributed (Figure 2, panel A).

This study and previous studies have increased understanding of *F. tularensis* subsp. *holarctica* in Europe by placing isolates from multiple countries into the existing global phylogeographic framework. As a result, the genetic background is becoming defined for each country (i.e., the specific subtypes reported from each country). This information can be useful for identifying intentional (e.g., bioterrorism) or unintentional movement of *F. tularensis* subsp. *holarctica* between countries. For example, the isolate from Romania examined in this study was actually isolated in Italy from an infected hare that was shipped from Romania for hunting. Genotyping results are consistent with a Romanian origin for this isolate because it was assigned to the B.13 group that is widespread in central and eastern Europe (Figure 2, panel A) and not to the B.FTNF002–00 group, to which the isolates from Italy were assigned (Figure 1, panel A).

Understanding global phylogeographic patterns is possible only if isolates from multiple geographic locations are placed within the same framework (i.e., examined with the same genomic signatures). Because *F. tularensis* is genetically monomorphic and highly clonal, SNPs are preferred signatures for determining phylogenetic structure within this species (*3*). Vogler et al. (*5*) conducted the first SNP-based global phylogeographic analysis of *F. tularensis*. Subsequent studies (*6**–**8*) have used the SNP signatures described by Vogler et al. (*5*) and new SNPs discovered from new whole-genome sequences or multiple sequence typing data to further refine phylogeographic patterns within *F. tularensis*, particularly *F. tularensis* subsp. *holarctica*. These new signatures, when screened across diverse isolate collections, have identified new subclades within preexisting subclades. This pattern will continue as whole-genome sequencing becomes less expensive and more widely available. As a result, the nomenclature of phylogenetic groups within *F. tularensis* and the particular subclade to which a given isolate is assigned are constantly changing and will continue to change, which makes comparison of results and findings across different studies difficult. To address this problem, we have included all known *F. tularensis* subsp. *holarctica* SNP-based phylogenetic groups within our phylogenetic trees (Figure 1, panel A; Figure 2, panel B), including those discovered by other researchers. In addition, for the isolates analyzed in this study (Table A1), where applicable, we have listed the phylogenetic groups to which they were assigned in previous studies.

## Appendix

**Table A1:** Isolates in study of the phylogeography of *Francisella tularensis* subsp. *holarctica*, Europe*

ID no.†	Original ID no.‡	Originating laboratory	Country of origin	Source§	Year	Subclade from (*5*)¶#	Subclade from (*8*)¶ **	Subclade from this study¶
F0586	AF19	BIM	Austria	*Lepus europaeus*	1997	13/14	20/21	33/34
F0587	AF9	BIM	Austria	*L. europaeus*	1996	13/14	20/21	33/34
F0589	AF6	BIM	Austria	*L. europaeus*	1994	13/14	20/21	33/34
F0590	AF5	BIM	Austria	*L. europaeus*	1997	13/14	20/21	33/34
F0591	AF3	BIM	Austria	*L. europaeus*	1997	13/14	20/21	33/34
F0599	AF22	BIM	Austria	*L. europaeus*	1997	13/14	20/21	33/34
F0595	AF54	BIM	Austria	*L. europaeus*	1994	13/14	20/21	34/35
F0597	AF36	BIM	Austria	Human	1997	13/14	20/21	34/35
F0588	AF8	BIM	Austria	*L. europaeus*	1995	13/14	20/21	36/37
F0614	DF161	BIM	Germany	Human	2007	13/14	20/21	33/34
F0616	DF159	BIM	Germany	*L. europaeus*	2007	13/14	20/21	33/34
F0620	DF155	BIM	Germany	Human	2007	13/14	20/21	33/34
F0626	DF163	BIM	Germany	Human	2007	13/14	20/21	33/34
F0627	DF162	BIM	Germany	Human	2007	13/14	20/21	33/34
F0634	DF170	BIM	Germany	*L. europaeus*	2007	13/14	20/21	33/34
F0639	DF193	BIM	Germany	*L. europaeus*	2008	13/14	20/21	33/34
F0640	DF194	BIM	Germany	*L. europaeus*	2008	13/14	20/21	33/34
F0641	DF195	BIM	Germany	*L. europaeus*	2008	13/14	20/21	33/34
F0642	DF196	BIM	Germany	*L. europaeus*	2008	13/14	20/21	33/34
F0643	DF197	BIM	Germany	*L. europaeus*	2008	13/14	20/21	33/34
F0601	DF101	BIM	Germany	*Macaca fascicularis*	2002	13/14	20/21	34/35
F0603	DF99	BIM	Germany	*M. mulatta*	2005	13/14	20/21	34/35
F0611	DF107	BIM	Germany	*M. fascicularis*	2005	13/14	20/21	34/35
F0617	DF158	BIM	Germany	*L. europaeus*	2007	13/14	20/21	34/35
F0621	DF169	BIM	Germany	*L. europaeus*	2007	13/14	20/21	34/35
F0625	DF164	BIM	Germany	*L. europaeus*	2007	13/14	20/21	34/35
F0704	T1	SZIU	Hungary	*L. europaeus*	2007	13/14	23/14/25	23/14/25
F0713	T17	SZIU	Hungary	*L. europaeus*	2008	13/14	20/21	20/21/33
F0714	T19	SZIU	Hungary	*L. europaeus*	2008	13/14	20/21	20/21/33
F0702	TM1	SZIU	Hungary	*Chlorocebus aethiops*	2003	13/14	20/21	33/34
F0703	TM2	SZIU	Hungary	*Erythrocebus patas*	2003	13/14	20/21	33/34
F0705	T3	SZIU	Hungary	*L. europaeus*	2007	13/14	20/21	33/34
F0706	T4	SZIU	Hungary	*L. europaeus*	2007	13/14	20/21	33/34
F0707	T6	SZIU	Hungary	*L. europaeus*	2007	13/14	20/21	33/34
F0709	T11	SZIU	Hungary	*L. europaeus*	2007	13/14	20/21	33/34
F0710	T12	SZIU	Hungary	*L. europaeus*	2007	13/14	20/21	33/34
F0711	T13	SZIU	Hungary	*L. europaeus*	2007	13/14	20/21	33/34
F0712	T14	SZIU	Hungary	*L. europaeus*	2008	13/14	20/21	33/34
F0716	T22	SZIU	Hungary	*L. europaeus*	2008	13/14	20/21	33/34
F0715	T21	SZIU	Hungary	*L. europaeus*	2008	13/14	20/21	34/35
F0708	T7	SZIU	Hungary	*L. europaeus*	2007	13/14	20/21	TUL07/2007
F0732	42055/2008	IZSLER	Italy	Natural spring water	2008	FTNF002–00	FTNF002–00	FTNF002–00
F0733	21851/2006	IZSLER	Italy	*L. europaeus*	2006	FTNF002–00	FTNF002–00	FTNF002–00
F0734	5768/2001	IZSLER	Italy	Human	2001	FTNF002–00	FTNF002–00	FTNF002–00
F0731	8660/1995	IZSLER	Romania/ Italy	*L. europaeus*	1995	13/14	20/21	33/34
F0433	Fr014	AFSSA	Central Europe††	*L. europaeus*	UNK	13/14	20/21	33/34
F0459	Fr040	AFSSA	Central Europe	*L. europaeus*	UNK	13/14	20/21	37/38
F0188	FSC 181	SDRA	Czech Republic	*Dermacentor reticulatus*	1995	13/14	20/21	33/34
F0190	FSC 183	SDRA	Czech Republic	*D. reticulatus*	1995	13/14	20/21	33/34
F0191	FSC 184	SDRA	Czech Republic	*D. reticulatus*	1995	13/14	20/21	33/34
F0193	FSC 186	SDRA	Czech Republic	*D. reticulatus*	1995	13/14	20/21	33/34
F0194	FSC 187	SDRA	Czech Republic	*Ixodes ricinus*	1995	13/14	20/21	33/34
F0187	FSC 180	SDRA	Czech Republic	*D. reticulatus*	1995	13/14	20/21	34/35
F0189	FSC 182	SDRA	Czech Republic	*D. reticulatus*	1995	13/14	20/21	34/35
F0192	FSC 185	SDRA	Czech Republic	*D. reticulatus*	1995	13/14	20/21	34/35
F0035	FSC 080	SDRA	Finland	*L. europaeus*	1984	13/14	20/21	20/21/33
F0163	FSC 249	SDRA	Finland	Human	1995	13/14	20/21	20/21/33
F0025	FSC 121	SDRA	Russia	Water	1985	13/14	20/21	20/21/33
F0026	FSC 123	SDRA	Russia	Water	1983	13/14	20/21	20/21/33
F0030	FSC 151	SDRA	Russia	Water	1988	13/14	20/21	20/21/33
F0034	FSC 077	SDRA	Sweden	*L. europaeus*	1981	13/14	20/21	20/21/33
F0040	FSC 188	SDRA	Sweden	Human	1996	13/14	20/21	20/21/33
F0093	FSC 102	SDRA	Sweden	Human	1981	13/14	20/21	20/21/33
F0227	FSC 273	SDRA	Sweden	Human	2000	13/14	20/21	20/21/33
F0302	FSC 175	SDRA	Sweden	Human	1995	13/14	20/21	20/21/33
F0036	FSC 081	SDRA	Sweden	*L. europaeus*	1985	13/14	20/21	33/34
F0226	FSC 272	SDRA	Sweden	Human	2000	13/14	20/21	33/34
F0209	FSC 248	SDRA	Sweden	Human	2000	13/14	20/21	35/36

*ID, identification; BIM, Institut für Mikrobiologie der Bundeswehr; SZIU, Szent István University; IZSLER, Istituto Zooprofilattico Sperimentale della Lombardia e dell’Emilia Romagna; AFSSA, Agence Française de Sécurité Sanitaire des Aliments and Laboratoire d'Etudes et de Recherches en Pathologie Animale et Zoonoses; UNK, unknown; SDRA, Swedish Defense Research Agency. †Isolate ID in the Northern Arizona University DNA collection. ‡Isolate ID from the originating laboratory. §*Lepus europaeus*, European brown hare; *Macaca fascicularis*, long-tailed macaque; *M. mulatta*, rhesus monkey; *Chlorocebus aethiops*, vervet monkey; *Erythrocebus patas*, patas monkey; *Ixodes ricinus*, castor bean tick (sheep tick); *Dermacentor reticulatus*, marsh tick. ¶In the interest of space, subclade names have been truncated to remove the preceding B.Br. nomenclature because all of these subclades belong in the B clade of *F. tularensis*. #Canonical single nucleotide polymorphism (canSNP) subclade originally defined in (*5*) and modified as described in the text. **CanSNP subclade defined in (*8*). These names have been modified to conform to the nomenclature standard originally established by (*5*) and modified as described in the text. ††Country unknown.

**Table:** Melt-MAMA primers targeting canonical SNPs for 6 new phylogenetic branches in a study of *Francisella tularensis* subsp. *holarctica*, Europe*

SNP	SCHU† S4 position	SNP state, D/A‡	Primers, 5′ → 3′§	Con, µM¶	T_m_, °C
B.33	78,382	T/C	A: ATTGCTACTTCTATTTACGCCAACAG	0.20	74.3
			D: GGGGCGGGGCGGGGCATTGCTACTTCTATTTACGCCAAGAA	0.20	79.0
			C: TGTGAACAACCAAGTTGGCTT	0.20	
B.34	766,614	A/G	A: GTAGCGAGCATTATTTGCTGGTTC	0.40	69.2
			D: GGGGCGGGGCGGGGCTAGCGAGCATTATTTGCTGGGTT	0.20	78.6
			C: ATAAAACTATAAATTTACATAAAATGAAAACTTCTC	0.20	
B.35	239,479	A/C	A: GCCTTAATCTAGTATTTTCGCTTATCTCC	0.40	70.3
			D: GGGGCGGGGCGGGGCGCCTTAATCTAGTATTTTCGCTTATCACA	0.20	75.5
			C: CGGGCTCTAAAATAAGATTTAAGTTAGTAAGT	0.20	
B.36	1,599,292	A/C	A: TATTATAGTTTCTAAAAACAGTCTAATTAATTTTG	0.60	69.0
			D: GGGGCGGGGCGGGGCTATTATAGTTTCTAAAAACAGTCTAATTAATTGTT	0.20	73.9
			C: GTTCGACCATGACTACAGTGTTG	0.20	
B.37	318,602	T/C	A: AACATTTTAGGAACTCTACGATGATAAACTTAAC	0.20	69.7
			D: GGGGCGGGGCGGGGCCATTTTAGGAACTCTACGATGATAAACTTGAT	0.20	75.9
			C: GAAATATCTCAATGAAATCTAATTTAACTAAAATCAC	0.20	
B.38	166,885	C/T	A: ATGCCATCAGCCATTTACTACTCACA	0.20	73.7
			D: GGGGCGGGGCGGGGCCCATCAGCCATTTACTACTCCCG	0.20	80.1
			C: CTTCCCTGATTTTCTAAGTTCTGCTTG	0.20	

*Melt-MAMA, melt-mismatch amplification mutation assay; SNP, single nucleotide polymorphism; con, concentration; T_m_, melting temperature for ancestral and derived Melt-MAMA amplification products. †SCHU strain GenBank accession no. NC_006570. ‡SNP states are presented according to their orientation in the SCHU S4 reference genome (AJ749949.2): D, derived SNP state; A, ancestral SNP state. §D, derived allele primer; A, ancestral allele primer; C, common primer; primer tails and antepenultimate mismatch bases are in lower case. ¶Final concentration of each primer in Melt-MAMA genotyping assays.
